# Identifying the conundrums of “global health” in the Global North and Global South: a case for Sub-Saharan Africa

**DOI:** 10.3389/fpubh.2024.1168505

**Published:** 2024-01-15

**Authors:** Luchuo Engelbert Bain, Oluwafemi Atanda Adeagbo, Cephas K. Avoka, Hubert Amu, Peter Memiah, Ikenna D. Ebuenyi

**Affiliations:** ^1^Department of Psychology, Faculty of Humanities, University of Johannesburg, Johannesburg, South Africa; ^2^International Development Research Centre (IDRC), Ottawa, ON, Canada; ^3^Department of Community and Behavioral Health, College of Public Health, University of Iowa, Iowa City, IA, United States; ^4^Department of Sociology, University of Johannesburg, Johannesburg, South Africa; ^5^Faculty of Public Health, Ghana College of Physicians and Surgeons, Accra, Ghana; ^6^Population and Behavioural Sciences, University of Health and Allied Sciences, Hohoe, Ghana; ^7^Division of Epidemiology and Prevention, University of Maryland School of Medicine, Baltimore, MA, United States; ^8^Department of Rehabilitation Science and Technology, University of Pittsburgh, Pittsburgh, PA, United States

**Keywords:** equity, global health, power, roles, solidarity

## Is global health public health? Recent global examples

The concept of “global health” is entrenched in the Western ideology of who is a human and the categorization of humanity. This categorization is ever-present in knowledge exchange and production, power relationships, and prioritization of health resources and allocation (1, 2). The most recent example is the “Global North” responses to the coronavirus (COVID-19) pandemic in low- and middle- income countries (LMICs), especially in Africa. In early 2020, two French scientists, dehumanized Africans and suggested that Africans should be used as “guinea pigs” for the COVID-19 vaccine trials despite the then relatively low COVID-19 morbidity and mortality in Africa (2, 3). This raised important questions about the perceived health equity, priority setting, and justice in “global public health” research. Indeed, the rationale for this raised serious ethical questions. If “global health is truly public health”, why is the life of a European considered more valuable than that of an African in this context? The aim of this commentary is to highlight the inequity in global health in the Global North and South with a specific focus on Africa.

At the peak of the COVID-19 pandemic, scapegoating and labeling (e.g., Chinese Virus, or South African Virus) of some LMICs was common (2, 3). For example, some African countries such as South Africa and Botswana were blamed for a variant (omicron) of the coronavirus which immediately shifted attention from the root causes of the pandemic. The United Kingdom, and other high-income countries (HICs) labeled and denied citizens of some African countries' entry to their countries because the omicron variant was first reported in South Africa and Botswana. Similar situations occurred during the allocation of COVID-19 vaccines. Some HICs pre-ordered billions of doses of COVID-19 vaccines that led to meager supply to LMICs particularly Africa even though some African countries were ready to pay for them. Unfortunately, anecdotal evidence shows that some of these vaccines' doses expired within HICs, while COVID-19 patent or knowledge know-how had not been shared with Africa to enable production of vaccine locally, a strategy that would have reduced dependency on HICs for COVID-19 vaccines (4, 5). In fact, OXFAM international reported that Europe threw away 55 million COVID-19 vaccine doses at the end of February 2022. This was 25 million more than the 30 million doses they donated to Africa at that point (6). Also, the EU blocked proposals which would allow Africa to manufacture COVID-19 vaccines (6). Similarly, the Monkey pox vaccine research agenda became a priority action area only when cases started being increasingly registered in the Global North. The solidarity and justice ideals of global health are to be seriously questioned.

## Redefining “global health”

Global health is centered on ensuring equity, eliminating barriers, and addressing disparities in health across the world (7, 8). We must begin to unpack the true meaning of global health by understanding what these two words represent. For the latter (health) and going by the World Health Organization (WHO) definition, “Health is a state of complete physical, mental and social wellbeing and not merely the absence of disease or infirmity” (9). Globalization, which is the growing interdependence of populations, economies, and cultures, impacts healthcare both positively and negatively. Although the WHO definition of health is well integrated, the connection between this definition and the challenges faced by LMICs in the context of globalization needs to be better articulated. Global health, therefore, recognizes the increasing homogeneity of social, economic, environmental, and political determinants of health and how this impacts access to quality healthcare worldwide and the health of populations in general (10, 11). While it is difficult to achieve consensus on the dimensions of global health, epistemic justice, social justice, solidarity, and health equity lie at its core (8, 11).

The global health security and response to Ebola and COVID-19 pandemic are glaring examples that showed that one's believe in the good faith of “global health” may be naive and recipe for disappointments. Could global health be another *myth* at the center of public health? In a recent reflection on *Maladies of Empire by* Jim Downs, Horton reported how the significant contributions of key players like “enslaved Africans” was hidden (intentionally) in the field of epidemiology (12). This type of intentional lack of transparency may not be very different when it comes to the way the global prioritization, funding, and research agendas operate in global health. The following fundamental questions in some core areas of global health beg for answers when it comes to the real intentions of what is termed “global health”.

### Health research and epistemic justice

I. What is global health and who defines the global health agenda? When is a health condition considered a global health problem?
II. If we aver the proposition by King and Koski (7) that global health is public health, why do some health problems receive low priority even in certain areas, particularly in LMICs, though they fall within the tenets of global health?
III. Who leads the research and publication space in global health? There is a shared responsibility between funders, journal editors, and scientists in the Global North, who have always occupied prestigious positions in “global health” publications, even when LMIC researchers and data collectors (including those with advanced degrees) did most of the work, and are times, even if rarely acknowledged.
IV. Why is research ethics permission from sites in LMICs often ignored? It is not uncommon for research led by researchers from the Global North to ignore or avoid research ethics for studies in the Global South (1). The lack of commitments to international standards of ethical research principles for study sites in the Global South leaves much to be desired!

### Equity in partnerships, power dynamics and decision-making

I. How many institutes (centers) of global health or postgraduate programs in global health are hosted in LMICs? The asymmetry is worrisome. For instance, it would be mutually beneficial to set up global health centers of excellence for tuberculosis (TB), HIV, and malaria in Africa than from the United Kingdom or elsewhere in HICs. Currently, most of the prestigious global health centers are based in HICs (2, 13).
II. The global health research agenda remains unclear. Although African countries were expected to be severely affected by COVID-19 at the early stage of the pandemic due to their vulnerabilities and weak health systems, the continent had one of the lowest COVID-19 related deaths (12). Despite the low COVID-19 mortality recorded in Africa, there is very little evidence to understand empirically why this happened, and any lessons that could be learned from this resistance that could apply elsewhere including HICs (1). We argue there seems to be an astute lack of transparency in global health policy and research agenda setting, with LMICs standing exclusively on a receiving end with fewer decision-making power.
III. If global health requires equity, then it has missed its mark. There is inequity in most global health partnerships and a lack of transparency.
IV. Funding for research, donor aid and economic empowerment: the asymmetry in power and funding pervades the global health space. Why is funding for projects in the Global South spent more on paying salaries of researchers and academics from the Global North (14)? Funders rely on an excellent research record as a pre-condition for awarding research funds. However, should the focus not be on capacity strengthening instead, since there is great disparity in capacity to produce excellent research, particularly in LMICs? It is saddening that the direct costs from most north - south research partnerships do end up exclusively in global north institutions.

Upon reflection, one is left to think that the concept of “global health”, “funding resources”, and application are part of a “hidden agenda” unknown to most LMICs scholars. It is tempting to question the real agenda of global health looking at the power dynamics with a focus on the main players from the Global North making key decisions that shape the landscape of global health worldwide. In global health, major plans and decisions continue to be made far away from where the actual problems and solutions are, despite many hitching a ride on the “country ownership” entitlement. The questions raised above are meant to guide reflection and discussion on whether global health is indeed public health for global good. Discussions on this issue should be centered on the current examples of disparities in global health, with the goal of ensuring equity continues to remain at the core of any global health agenda.

## Key recommendations

Transparency must constitute a key value in global health agenda setting. Valid and time-relevant propositions and measures must be adopted. Despite the issues facing global heath practices and ideals, equitable partnerships stand out as a core generally accepted ethical ideal in global health practice that must be upheld (14, 15). Deconstructing the various dimensions of power imbalance in global health is a needed starting point if truly a global health agenda that addresses global health disparities must be achieved. Equitable investments must be made when it comes to training in global health, where centers of excellence should be distributed equitably between the Global North and the Global South for mutual learning and knowledge exchange.

We are aware of the fact that similar challenges are faced in other low-income settings other than Africa. Contextually grounded reflections on the drivers of power imbalance as well as reflecting upon how colonialism impacts interactions between Global North and South actors remain critical. Dee and Lasco from the Philippines have nicely reported on how the global power, health knowledge and academic systems continue to function on purely colonial realms. Indeed, they have proposed acknowledgment and clear lines of action by former colonial powers in decolonizing global health, coming up with mutually agreed upon decolonized global health funding frameworks, and working on a more inclusive scholarly publishing and recognition agenda (15). Although this could apply to other low-income settings, our focus in this commentary is Africa. To address some of these issues, we join the call for the decolonization of global health and more active research, symposia, and funding to establish global health research centers in LMICs (particularly Africa), where most global health-related research is conducted. Academic and non-academic actors need to urgently work together to dismantle the current structure of global health that mainly favors the Global North. If global health is public health, it should depict global solidarity and justice, that promotes equity in the distribution of health-related resources including funding and knowledge production and seek to co-creatively close health disparity across the globe. It is imperative that research agenda setting for global health initiatives consider national needs and priorities in the Global South in consultation with local actors in the settings for the interventions. We acknowledge that some countries in Africa are partly responsible for this power imbalance due to factors related to corruption, weak research governance architectures, and insignificant investments in research. Yet, we advocate for more transparency in global health governance and structure that further entrenches equity in order to drive real health development in the global South.

## Author contributions

LB and IDE conceived the idea and wrote the initial draft. OA and LB provided astute intellectual input that led to the final draft of the paper. IDE provided overall direction of the writing process. CA, HA, and PM provided core ideas and critically reviewed the manuscript. All authors have agreed on the final version of the manuscript before submission.
